# Determination of the antiulcer properties of sodium cromoglycate in pylorus-ligated albino rats

**DOI:** 10.4103/0253-7613.66844

**Published:** 2010-06

**Authors:** Vivek Srivastava, A.H.M. Viswanathaswamy, Govind Mohan

**Affiliations:** Department of Pharmacology, KLE Society College of Pharmacy, Hubli, India; 1 Department of Pharmacology, S.N. Medical College, Agra, India

**Keywords:** Antiulcer activity, pyloric ligation model, sodium cromoglycate, ulcer index

## Abstract

**Objectives:**

To study the ulcer protective property of sodium cromoglycate in pylorusligated rats and the biochemical role in ulcer protection by various biochemical tests.

**Materials and Methods:**

The ulcer protective effect of sodium cromoglycate was studied using a Pyloric Ligation Model using Wistar albino rats. The antiulcer effect of sodium cromoglycate 40 mg/kg b.w., i.p., was compared with the reference drug ranitidine 27 mg/kg b.w., i.p. The ulcer index was calculated and other biochemical parameters like free acidity, total acidity, pH, mucin, pepsin and volume of gastric juice were determined.

**Results:**

Pylorus ligation showed a significant (*P* < 0.01) reduction in gastric volume, free acidity, total acidity and ulcer index as compared to the control.

**Conclusion:**

Sodium cromoglycate has activity equipotent to ranitidine.

## Introduction

Gastric ulcers arise due to net imbalances in mucosal offensive and defensive factors.[1] Ulcer therapy is now mainly focused on limiting the deleterious effects of offensive acid secretion, but the search for newer, safer drugs have rekindled the interest in drugs that protect the gastric mucosa from damaging agents without influencing acid secretion or neutralizing intragastric acidity. It is well known that the gastric mucosa can resist autodigestion although it is exposed to numerous noxious stimuli like aggressive secretions of hydrochloric acid, pepsin, reflex of bile, spicy food, microorganisms, formation of free radicals, stress, alcohol, 5-hydroxy tryptamine, substance P (SP), slow releasing substance, irritant receptors and platelet activating factor.

The imbalance between offensive factors and defensive factors may contribute to the formation of gastric ulceration. Drugs either inhibit the offensive factors or boost the defensive factors that are the mechanisms for antiulcerogenic activities. Sodium cromoglycate (cromolyn, disodium cromoglycate [DSCG]) is extensively used in the management of asthma,[2] allergic rhinitis, persistent diarrhea,[3] vernal conjunctivitis, aphthous stomatitis and eczema. It is also a useful adjunct in the treatment of ulcerative colitis.[4] Sodium cromoglycate being a mast cell stabilizer[5] inhibits the Ca ^2+^ -mediated peptic ulcers, histamine and leukotrienes as well as SP-impaired hyperemic response mediated by calcitonin gene-related peptide[6] (CGRP). Pre-treatment with sodium cromoglycate[7] appears to protect against the further deregulation of mast cells caused by SP and restoration of hyperemic response after mucosal injury and acid challenge. DSCG inhibits the release of chemical mediators and has been used for the prevention of allergic diseases such as asthma.[8,9] Recently, the inhibitory effect of DSCG on influenza virus[10] and duodenal ulcer[11] has been reported. Therefore, the present study has been conducted to investigate the gastric antiulcer properties of sodium cromoglycate in albino rats.

## Materials and Methods

Wistar albino rats of either sex with an average body weight of 100 g were selected for the present study. All the reagents used in the biochemical analysis were procured from Ranbaxy and SD Fine Chemicals, (New Delhi, India). Pure drug samples of sodium cromogylate and ranitidine were procured from Cipla Pharmaceuticals. A Hitachi 15-20 (Hitachi, Japan)spectrophotometermodel was used for the biochemical studies. Animal studies were performed with prior permission of Institutional Animal Ethics Committee (Reg. No. 126/1999/CPCSEA under the rule 5(a) of the “Breeding of and experiments on animals [control and supervision] rules 1998”).

**Figure 1:**
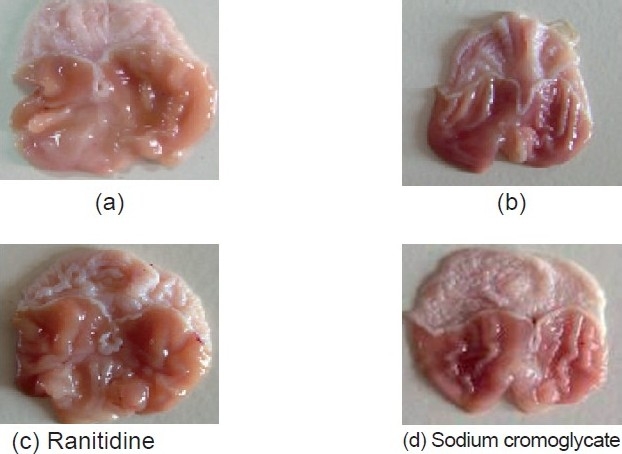
Comparative antiulcer study of ranitidine and sodium cromoglycate

The ulcer protective effect of sodium cromoglycate was measured as per the method of Shay *et al*.[12] In this method, albino rats were fasted in individual cages for 24 h. Control vehicle, sodium cromoglycate[13] (40 mg/kg, i.p.), and reference drug, ranitidine (27 mg/kg, i.p.), were administered. The pyloric ligation was carried out 30 min and 4 h after the drug administration in animals of each group. Light ether anesthesia was given and the abdomen was opened subsequently with the ligation of the pylorus. The abdomen was then sutured. After 4 h of pyloric ligation, the animals were sacrificed with excess of anesthetic ether and the stomach was dissected. Gastric juice was collected and its volume, pH and total acidity were measured. Ulcer index was determined. The glandular portion of the stomach was opened along the greater curvature and the severity of hemorrhagic erosions in the acid secreting mucosa was assessed on a scale of 0–3 [Figure 1]. Results of the study are summarized in Table 1. The percentage protection was calculated using the following formula:

**Table 1:** Antiulcer effect of sodium cromoglycate (cromolyn) in the pylorustreated model

*Sr. no*	*Treatment*	*Ulcer index*
1	Control	5.5833 ± 0.2713
2	Ranitidine	2.0 ± 0.3162*
3	Cromolyn	2.25 ± 0.3354*
F, df		41.970 (2/15)
*P*-value		*P* < 0.0001

Values are mean ± SEM, n = 6

* *P* <0.01 when compared with control

Percentage of ulcer protection = Ut / Uc × 100

where,

Ut = ulcer index of treated group, and

Uc = ulcer index of control group

### Biochemical Estimations

Gastric juice was collected from pylorus-ligated rats. The gastric juice thus collected was centrifuged and the volume of gastric juice as well as the pH of gastric juice was noted. The gastric juice was subjected to biochemical estimations as follows:

### Determination of free and total acidity[14]

One milliliter of gastric juice was pipetted into a 100 ml conical flask, two or three drops of Topfer’s reagent was added and this was titrated with 0.01 N sodium hydroxide until all traces of red color disappeared and the color of the solution became yellowish-orange. The volume of alkali added was noted. This volume corresponds to free acidity. Two or three drops of phenolphthalein solution was added and titration was continued until a definite red tinge appeared. The total volume of alkali added was noted. The volume corresponds to total acidity. Acidity was calculated using the following formula:

$$\mathrm{Acidity}\;=\;\frac{\mathrm{Volume}\;\mathrm{of}\;\mathrm{NaOH}\;\times \;\mathrm{Normality}\;\mathrm{of}\;\mathrm{NaOH}\;\times \;100}{0.1}\mathrm{meq}/\mathrm{Lit}/100\;\mathrm{gm}$$

Statistical significance was determined using Student’s *t*-test and ANOVA [Table 2].

**Table 2:** Estimation of pH, free acidity and total acidity of gastric juice

*Sr. no*.	*Treatment*	*Volume of gastric juice (ml)*	*pH*	*Free acidity (meq/l/100 g)*	*Total acidity (meq/l/100 g)*
1	Control	3.15 ± 0.0846	1.75 ± 0.07638	21.66 ± 1.944	82.0 ± 2.176
2	Ranitidine	1.21 ± 0.05426*	2.9 ± 0.1065*	9.5 ± 0.4282*	38.33 ± 1.430*
3	Cromolyn	1.45 ± 0.1565*	2.6 ± 0.1033*	12.6 ± 0.5578*	51.1 ± 1.167*
F, df value		97.001 (2/15)	38.334 (2/15)	28.028 (2/15)	185.77 (2/15)
*P*-value		<0.0001	<0.0001	<0.001	<0.0001

Values are mean ± SEM, n = 6

* *P* < 0.01 when compared with control

### Estimation of total proteins[15]

The dissolved proteins in gastric juice was estimated in the alcoholic precipitate obtained by adding 90% alcohol with gastric juice in a 9:1 ratio. Then, 0.1 ml of the alcoholic precipitate of gastric juice was dissolved in 1 ml of 0.1 N NaOH and, from this, 0.05 ml was taken into another test tube. To this, 4 ml of alkaline mixture was added and kept for 10 min. After a while, 0.4 ml of phenol reagent was added and after 10 min color started developing again. A reading was taken against blank prepared with distilled water at 610 nm using a Hitachi 15-20 spectrophotometer. The protein content was calculated from the standard curve prepared with bovine albumin and expressed as micrograms/milliliter of gastric juice [Table 3]. The statistical analysis was performed using Student’s *t*-test.

**Table 3:** Estimation of mucin, total protein and pepsin activity

*Sr. no*	*Treatment*	*Gastric wall mucus content (μg of alcian blue/g of wet gland)*	*Total protein (μg/ml)*	*Pepsin activity (μmole/ml*
1	Control	232.66 ± 6.064	100.83 ± 1.108	42.49 ± 0.8044
2	Ranitidine	309.66 ± 4.248*	95.33 ± 2.404†	36.705 ± 1.486*
3	Cromolyn	304 ± 6.470*	92.5 ± 0.5627*	32.48 ± 1.104*
F, df value		65.306 (2/15)	7.349 (2/15)	18.595 (2/15)
P-value		<0.0001	<0.006	<0.0001

Values are mean ± SEM,*n* = 6

* *P* < 0.01 when compared with control;

† *P* < 0.001 when compared with control

### Estimation of mucin[16]

After the collection of gastric juice, the glandular portion excisions that opened the lesser curvature were opened. The everted stomachs were soaked for 2 h in 0.1% alcian blue 8GX dissolved in 0.16 M sucrose buffered with 0.05 M sodium acetate adjusted to a pH with hydrochloric acid. Uncomplexed dye was removed by two successive washes of 15 and 45 min in 0.25 M sucrose solution. Dye complex with mucus was diluted by immersion in 10 ml aliquots of 0.5 M magnesium chloride for 2 h. The resulting blue solutions were shaken briefly with an equal volume of diethyl ether and the optical density of the aqueous phase was measured at 605 nm using a Hitachi 15-20 spectrophotometer. The mucin content of the sample was determined [Table 3] from the standard curve of mucin, which has been expressed in microgram/gram of wet gland tissue.

### Estimation of pepsin[17]

Aliquots of 20 *μ*l of the gastric content were incubated with 500 *μ*l of albumin solution (5 mg/ml in 0.06 N hydrochloric acid) at 37C for 10 min. The reaction was stopped with 200 *μ*l of 10% trichloroacetic acid and the samples were centrifuged at 1500 g for 20 min. The supernatant was alkalinized with 2.5 ml of 0.55 M sodium carbonate and 400 *μ*l of 1.0 N Folin’s reagent was added to the tubes, which were incubated for 30 min at room temperature. The absorbance of the samples was determined by spectrophotometry at 660 nm. The concentration of pepsin is determined by a standard curve.

## Results

In the present study, an attempt has been made to investigate the gastric antisecretory, antiulcer and cytoprotective properties of sodium cromoglycate. It is evident from Table 1 and Figure 1 that the ulcer index of sodium cromoglycate is comparable with ranitidine against the control. The results are statistically significant by ANOVA test. Ranitidine showed a decrease in the volume of gastric juice by 61.5%, free acidity 51.6% and total acidity 53.2% when compared to control, which is statistically significant [Table 2]. Sodium cromoglycate shows a decrease in the volume of gastric juice by 53.9%, free acidity 41.8% and total acidity 37.6%, which is also statistically significant. A significant difference in pH was observed between the sodium cromoglycate-treated and the control groups.

## Discussion

Ulcer is a recurrent disease affecting large populations in all geographical regions, and reactive oxygen species have been implicated in the pathogenesis of a wide variety of clinical disorders and gastric damage. Peptic ulcers result from an imbalance between defensive (cytoprotective) and offensive factors (gastric acid), association with *Helicobacter pylori* infection and increased use of non-steroidal anti-inflammatory drugs like aspirin and indomethacin,[18] causing damage by inhibiting the biosynthesis of cytoprotective prostaglandins.[19]

In the present study, the significant reduction in basal gastric secretion and complete inhibition of ulcers by sodium cromoglycate after pylorus ligation suggests that its cytoprotective mechanism of action on the gastric mucosa may be responsible for the direct reduction of gastric secretion through one or more of the possible mechanisms discussed by Martin *et al*.[20] Gastric acid secretion is regulated by many factors including anxietic effect in the central nervous system, vagal activity, irritant receptors and histaminergic and gastrinergic neurotransmissions, including the proton pump. The current data clearly demonstrated that sodium cromoglycate inhibited the aggressive factor and gastric acid secretion. The antiulcerogenic effect of sodium cromoglycate may be related to its antisecretory action because acid is a major factor in the development of peptic ulcer. The results obtained from the present studies revealed that sodium cromoglycate has anticholinergic[21] and vagolytic activities.[22] It also shows a prompt inhibitory effect on the irritant receptors. The antisecretory and antiulcerogenic activity of sodium cromoglycate observed in the present study is in agreement with earlier reports.[23,24] However, certain antiulcer drugs increase the amount of gastric mucus secretion in the gastric mucosa. The mucus consists of mucin-type glycoproteins, which can be detected by amounts of alcian blue binding.[16]

Thus, the possible mechanism of gastric mucosal protection by sodium cromoglycate may be partly due to the reinforcement of resistance of the mucosal barrier by a protective coating. Sodium cromoglycate has shown increased pH and decreased total acidity of gastric fluid. The antiulcer effect was also supported by a decrease in aggressive factors like pepsin and an increase in defensive factors like mucin. The decrease in the protein content of gastric juice by cromoglycate suggests a decrease of leakage of plasma proteins into gastric juice. This also suggests an increase in the glycoprotein content of the gastric mucosa, acting as a coating and protective barrier on the gastric mucosa.

## Conclusion

From the above study, it was found that when compared with ranitidine, sodium cromogylate showed an equipotent effect on the pylorus ligation model. A significant increase in the mucin content, decreased total proteins and pepsin content in rats treated with sodium cromoglycate shows strong evidence for the antiulcer activity of the drug.
